# The Utility of Fluorescence Recovery after Photobleaching (FRAP) to Study the Plasma Membrane

**DOI:** 10.3390/membranes13050492

**Published:** 2023-05-02

**Authors:** Charles A. Day, Minchul Kang

**Affiliations:** 1 Hormel Institute, University of Minnesota, Austin, MN 55912, USA; 2 Mayo Clinic, Rochester, MN 55902, USA; 3 Department of Mathematics, Texas A&M—Commerce, Commerce, TX 75428, USA; minchul.kang@tamuc.edu

**Keywords:** fluorescence recovery after photobleaching, FRAP, diffusion, plasma membrane, microdomain, actin, confocal microscopy

## Abstract

The plasma membrane of mammalian cells is involved in a wide variety of cellular processes, including, but not limited to, endocytosis and exocytosis, adhesion and migration, and signaling. The regulation of these processes requires the plasma membrane to be highly organized and dynamic. Much of the plasma membrane organization exists at temporal and spatial scales that cannot be directly observed with fluorescence microscopy. Therefore, approaches that report on the membrane’s physical parameters must often be utilized to infer membrane organization. As discussed here, diffusion measurements are one such approach that has allowed researchers to understand the subresolution organization of the plasma membrane. Fluorescence recovery after photobleaching (or FRAP) is the most widely accessible method for measuring diffusion in a living cell and has proven to be a powerful tool in cell biology research. Here, we discuss the theoretical underpinnings that allow diffusion measurements to be used in elucidating the organization of the plasma membrane. We also discuss the basic FRAP methodology and the mathematical approaches for deriving quantitative measurements from FRAP recovery curves. FRAP is one of many methods used to measure diffusion in live cell membranes; thus, we compare FRAP with two other popular methods: fluorescence correlation microscopy and single-particle tracking. Lastly, we discuss various plasma membrane organization models developed and tested using diffusion measurements.

## 1. Introduction

The plasma membrane is a highly complex and actively regulated system that controls various cellular functions, including cell surface attachment and motility, receptor-mediated signaling, endocytosis, and exocytosis. To understand how the cell membrane regulates these processes, we must know the basic architecture of the cell surface. The highly dynamic and nanoscale organization of cell membranes limits the amount of information that can be gathered from conventional fluorescence imaging techniques. Traditional fluorescence microscopy techniques, such as widefield microscopy, total internal reflection fluorescence microscopy, and confocal microscopy, do not have the resolution to image much of this complexity directly. In addition, super-resolution techniques are not readily accessible and often rely on long-term imaging, limiting the information gathered on a highly dynamic system, such as the plasma membrane.

Consequently, to study the subresolution organization of live cell membranes, cell biologists have turned to biophysical approaches that report on the physical properties of the plasma membrane to infer structure. These techniques can be grouped into those that report on molecular clustering (such as Förester resonance energy transfer (FRET) and fluorescence-lifetime imaging microscopy (FLIM)) and those that measure diffusion. Among the techniques that measure diffusion, the most common are single-particle tracking, fluorescence correlation spectroscopy, and fluorescence recovery after photobleaching. Each of these techniques has its strengths and weaknesses. 

Our modern understanding of the plasma membrane started primarily in 1970, when Frye and Edidin, in their groundbreaking work, demonstrated that some components of the plasma membrane are not static but highly dynamic [1]. In those experiments, cells were marked with differentially labeled antibodies against membrane proteins. The cells were then fused. Time-lapse imaging showed that the proteins from the two cells mixed over time, proving that a population of cell membrane proteins is mobile. That finding was instrumental in Singer and Nicolson proposing the Fluid Mosaic model just two years later [2]. Because molecular diffusion is governed by the physical properties of both the diffusing molecule and the surrounding media, the discovery that the plasma membrane is fluid opened the door to studying the plasma membrane through diffusion measurements. 

In 1905, Albert Einstein and William Sutherland, separately, described diffusion in three dimensions as

(1) $$D=\frac{k_{B}T}{6\pi \eta r}$$

where *D* is the rate of diffusion, *k_B_* is the Boltzmann constant, *T* is absolute temperature, and η is the viscosity of the media. In this equation, commonly known as the Stokes–Einstein equation, the diffusing particle is assumed to be spherical, and its hydrodynamic radius is represented as *r* [3,4]. 

Saffman and Delbruck revised the Stokes–Einstein equation in 1975 to restrict diffusion to a two-dimensional plane [5]. Their equation,

(2) $$D=\frac{k_{B}T}{4\pi \eta h}\left(\log\frac{\eta h}{\eta^{\prime }r}-0.5572\right)$$

expands on the Stokes–Einstein equation by separating the membrane viscosity (η) from the viscosity of the media outside the membrane ($\eta^{\prime }$), which is much smaller than the membrane viscosity. The membrane thickness is regarded as h. And r is the hydrodynamic radius of the diffusing particle, which is approximated as a cylinder traversing the membrane. Conceptually, Equation (2) means that diffusion is directly proportional to temperature. Additionally, the diffusion rate will decrease as either the membrane’s viscosity or the diffusing particle’s size increases. 

The Saffman–Delbruck equation has been instrumental in two ways. First, it laid the groundwork for developing the equations needed to derive quantitative values from experimental data, such as determining a rate of diffusion from a FRAP recovery curve. Furthermore, the Saffman–Delbruck equation assumes diffusion in a simple, homogeneous environment. Yet, experimental data reveals that membrane components rarely display free diffusion consistent with a simple, homogeneous bilayer [6]. Therefore, examining cases where the experimental data doesn’t match the model set forward with the Saffman–Delbruck equation has led to a greater understanding of the plasma membrane, as discussed in detail in Section 4. 

The Saffman–Delbruck Equation is based on a model of “Free Diffusion”. In this model, diffusion is controlled by Brownian motion and encumbered only by the viscosity of the membrane. One of the features of free diffusion is that distance traveled and time are proportional across all scales. In other words, a particle’s rate of diffusion will be the same whether measured over one second or one minute, and the distance traveled in one minute will be 60 times that traveled in one second. At times, diffusion measurements in live cell membranes have revealed free diffusion. However, they have also shown diffusion behaving as anomalous super-diffusion or anomalous sub-diffusion. Super-diffusion is when the rate of travel increases the longer the molecule is measured. For example, if a particle travels one μm in one second but more than 60 μm in one minute, this behavior would be considered “super-diffusion”. Conversely, “sub-diffusion” describes a particle that slows with time and distance, such as a particle that might travel one μm in one second but less than 60 μm in one minute. How these forms of diffusion are detected experimentally are discussed in Section 2 and Section 3, and examples of cell membrane features that generate super-diffusion and sub-diffusion are discussed in Section 4. 

## 2. Basic FRAP Methodology and Data Analysis

### 2.1. FRAP Methodology 

**Figure 1:**
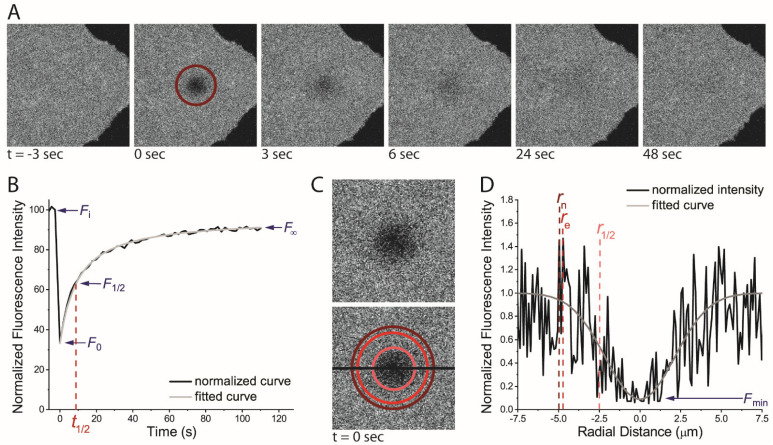
Basic Methodology of the FRAP Experiment and Data Analysis. (**A**) Time−lapse images of FRAP were performed on the plasma marker, Alexa546 tagged cholera toxin B subunit (A546-CTxB), labeling a COS-7 cell. Data were collected on a line-scanning confocal microscope at 37 °C. The dark red circle denotes the bleached region (i.e., the nominal bleach region). (**B**) Fluorescence recovery in the nominal bleach ROI from (**A**) that has been corrected for photodecay caused by imaging, normalized to a prebleach intensity of 100 and plotted over time. (**C**) Zoomed-in image of the first image after the bleach (t = 0 s) from (**A**). On the bottom, the nominal ROI is denoted in dark red, the effective radius (*r*_e_) in red, and the half max (*r*_1/2_) in pink. (**D**) Raw fluorescence intensity profile of cross section (dark line in panel **C**) through the bleach ROI.

Many detailed methods papers exist on the technical aspects of FRAP (See [7,8,9]). In brief, the FRAP experiment for observing diffusion in the plasma membrane begins by achieving a uniform (or nearly uniform) labeling of the plasma membrane with a fluorescence marker—be that a fluorescently tagged protein or a fluorescent lipid probe. A few pre-bleach images are first collected at low light levels on the microscope. Then, a small region of interest (ROI) is photobleached using a short-interval, high-intensity laser. Time-lapse imaging is resumed to film the photobleached ROI as fluorescent molecules from outside the ROI diffuse into the bleached region (Figure 1A). From the FRAP recovery curve, the fraction of mobile particles and the rate of diffusion for those mobile particles can then be derived. 

### 2.2. Calculating the Mobile Fraction 

One piece of information that can be easily acquired from the experimental FRAP data is the mobile fraction (or *M*_f_). The mobile fraction represents the percentage of the studied molecules free to move throughout the membrane. The mobile fraction can be derived easily by the equation,

(3) $$M_{f}=\left(\frac{F_{\infty }-F_{0}}{F_{i}-F_{0}}\right)$$

where $F_{\infty }$, $F_{0}$, and $F_{i}$ are the normalized fluorescence intensities inside the bleach ROI after full recovery (at the asymptote), immediately following the bleach, and before the bleach, respectively (Figure 1B). Before calculating the mobile fraction, it is important that the $F_{\infty }$, $F_{0}$, and $F_{i}$ are normalized for the photodecay from imaging and the loss of total cellular fluorescence from the photobleaching event. This is achieved by normalizing the entire FRAP curve using the equation

(4) $$F{\left(t\right)}_{norm}=\frac{F{\left(t\right)}_{ROI}-F_{bkgd}}{F{\left(t\right)}_{cell}-F_{bkgd}}\times \frac{F{\left(i\right)}_{cell}-F_{bkgd}}{F{\left(i\right)}_{ROI}-F_{bkgd}}$$

where $F{\left(t\right)}_{ROI}$ and $F{\left(t\right)}_{cell}$ are the intensities of the ROI and the whole cell, respectively, at each time point F(t). Similarly, $F{\left(i\right)}_{ROI}$ and $F{\left(i\right)}_{cell}$ are the intensities of the ROI and the whole cell at the start of the experiment. Additionally, $F_{bkgd}$ is fluorescence intensity outside the cell. 

### 2.3. Deriving a Diffusion Coefficient from FRAP Data

The rate of diffusion can also be acquired from the FRAP data. However, this is much more complicated than acquiring the mobile fraction, and various methods have been proposed for deriving the diffusion coefficient [10,11,12,13]. These methods differ in terms of accuracy and ease. 

A significant consideration when selecting a method for acquiring a diffusion coefficient is how the bleaching was performed, as different methods of photobleaching demand different mathematical regimes to obtain an appropriate diffusion coefficient. This is because with a static beam line, the photobleaching event can occur so quickly as to be considered instantaneous. However, in line-scanning confocal FRAP, the diffraction-limited laser beam is rastered over the ROI for the set number of cycles required to bleach the ROI adequately. Since this is not an instantaneous process, molecules near the edge of the ROI can diffuse into and out of the ROI during the photobleaching process. Calculating diffusion from data collected on a line-scanning confocal microscope cannot be carried out with a high degree of accuracy using equations created for static beam FRAP that do not account for diffusion during the bleaching event [12,14]. Furthermore, the faster the diffusion of the molecule being studied or the longer the bleaching event, the greater the error in the diffusion coefficient becomes [10,11]. 

In most instances, FRAP recovery can be well described by equations that assume that all molecules measured were diffusing by the same single mode. This would be the case when, for example, all the particles being examined undergo free diffusion as monomers. The single-component diffusion assumption has been sufficient to describe some diffusion, and we have restricted our discussion of data processing to deal with only single-component diffusion. However, single-component diffusion is not the perfect description of all diffusion within the plasma membrane. For example, some molecules have different populations undergoing different modes of diffusion simultaneously [15,16]. This could take many forms, such as a mixture of freely diffusing monomers and higher-order complexes.

#### 2.3.1. Deriving a Diffusion Coefficient Using the Half-Time of Recovery from a Static Bleaching Beamline FRAP

A simple approach to determining the diffusion coefficient is to use the half-time of recovery ($t_{1/2}$). The $t_{1/2}$ is the time point after the photobleaching event at which half of the mobile fluorescent molecules have diffused back into the ROI. This value is representative of recovery time and, thus, reflects the diffusion rate. The $t_{1/2}$ can be approximated by fitting Equation [17]

(5) F(t)=[F0−F∞(tt1/2)]/[1+(tt1/2)]

Once a $t_{1/2}$ value has been determined, a rate of diffusion (also known as the diffusion coefficient or *D*) can be approximated by the simple equation [14],

(6) $$D=0.224\frac{r_{n}^{2}}{t_{1/2}}$$

where $r_{n}$ (or nominal radius) is the radius of the bleach spot. It must be noted that this equation works only if the ROI is circular and would need altered if a different shape is used for the ROI [14].

#### 2.3.2. Deriving a Diffusion Coefficient by Modeling Recovery from a Static Bleaching Beamline 

A more complex method for determining the diffusion coefficient was proposed in 1976 by Axelrod and Webb [12]. Their approach involves modeling potential recovery curves using the equation,

(7) $$F\left(t\right)=\overset{\infty }{\underset{n=0}{\sum }}\frac{{\left(-K\right)}^{n}}{n!\left(1+n\left[1+2t/\tau_{D}\right]\right)}$$

where *K* is the bleach depth parameter and $\tau_{D}=r_{n}^{2}/\left(4D\right)$ is the diffusion time. When t=0, $F\left(0\right)=\left(1-e^{-K}\right)/K$, from which K can be determined numerically. The correct diffusion rate can be identified by modeling different curves with different *D* values and comparing them with the experimental recovery curve. 

#### 2.3.3. Deriving a Diffusion Coefficient by Modeling Recovery from Line-Scanning Confocal FRAP Data 

The protocol developed by Axelrod and Webb (Equation (7)) was created using a static beam of high-intensity laser light to perform photobleaching [12,18,19,20]. This approach works well for performing FRAP in a widefield or spinning disk confocal using a static bleach line, as the bleach can be treated as instantaneous. However, it significantly underreports diffusion when applied to FRAP data from a line-scanning confocal microscope [10,11]. 

Several equations have now been derived to correct for diffusion during the bleaching so that correct *D* values can be acquired from line-scanning confocal FRAP data [10,11,13]. One approach is to replace the nominal bleaching ROI radii with an altered ROI radius, referred to as the effective ROI (or *r_e_*). The *r_e_* can be determined by plotting the fluorescence intensity as a cross section of the ROI at the first time point after the photobleaching event (Figure 1D). Fitting that plot with

(8) $$f\left(r\right)=\left(1-K\exp\left(-\frac{2r_{n}{}^{2}}{r_{e}^{2}}\right)\right)$$

for K and $r_{n}$ allows $r_{e}$ to be solved. Alternatively, $r_{e}$ can be found from the half-width at the half-depth [21], and $r_{1/2}$ from the postbleach profile as

(9) $$r_{e}=r_{1/2}\sqrt{\frac{2}{\ln2}}≅1.7r_{1/2}$$

Once the effective radius is known, it can be used in a modified form of the Soumpasis equation (Equation (6)) to derive a diffusion coefficient [22], as follows:

(10) $$D=\frac{r_{e}^{2}+r_{n}^{2}}{8t_{1/2}}$$

Alternatively, a FRAP model for diffusion in membranes has been derived, which is better suited to FRAP data from confocal laser scanning microscopes by correcting artifacts, such as diffusion, during the photobleaching [22]

(11) $$F\left(t\right)=F_{i}\left\{1-\frac{K}{1+\gamma^{2}+2t/\tau_{D}}\right\}M_{f}+\left(1-M_{f}\right)F_{0}$$

where $\tau_{D}=r_{e}^{2}/\left(4D\right)$ and γ is $r_{n}/r_{e}$.

Knowing when to apply each mathematical regime is key to acquiring the most accurate diffusion coefficients. For example, Equations (6) and (7) are suitable for processing FRAP data collected on widefield microscopes or spinning disk confocal microscopes. However, Equations (10) and (11) will yield dramatically more accurate diffusion coefficients from line-scanning confocal FRAP data than Equations (6) and (7) [11]. Moreover, while Equations (5) and (9) are simpler than other methods, they rely heavily on a single point in the recovery curve (the $t_{1/2}$), so a minor fluctuation in the experimental data at $t_{1/2}$ can dramatically alter the result. Therefore, while curve-fitting (Equation (11)) is the most complicated method, it is also the most accurate method for calculating a diffusion coefficient from line-scanning confocal FRAP. 

The data analysis in which a FRAP recovery curve is modeled mathematically (Equations (7) and (11)) requires some method to evaluate the accuracy of that model to describe the experimental data. The best model to describe the data can be determined by the sum of the squared error (SSE) between the two curves, and then the model with the lowest SSE is used as the best representation of the experiment and, therefore, has the most accurate diffusion coefficient [7,11,23].

### 2.4. Variations on FRAP

A variation on the classical FRAP experiment that has been instrumental in membrane biophysics is the variable ROI FRAP (or “vrFRAP”). As its name suggests, this method involves collecting FRAP data from ROIs of different sizes. In the first application of this technique, the Edidin lab reported diffusion coefficients for a fluorescent lipid analog that could be easily separated into a fast or a slow population when FRAP was collected on a small spot size. However, when a larger spot size was used for FRAP on the same lipid analog, repeated experiments generated *D* values clustered into a single group—between the two separate diffusion coefficients measured at the small spot size. They hypothesized that their membrane marker was sampling two membrane regions with distinct diffusion characteristics, such as small, viscous domains surrounded by interconnected membrane regions. If FRAP were performed with an ROI smaller than the domain size, then the *D* value would represent either slow diffusion inside the domain or fast diffusion outside the domain—depending on where the bleach spot fell on the cell surface. However, using a bleach spot larger than the domain size would yield a *D* value that represented an average of diffusion inside and outside these domains [24]

This initial study and follow-up studies using vrFRAP (on live cells, supported bilayers, or in silico modeling [24,25,26,27]) have shown that plotting vrFRAP data vs. radius is a powerful tool for determining underlying membrane organization. For instance, the dependence of *M*_f_ on molecular confinement was nicely revealed by FRAP modeling from Salomé et al. [27]. They performed vrFRAP simulations where the diffusing particle underwent free or restricted diffusion in domains smaller than the bleaching ROI. Plotting *M*_f_ vs. 1/*r* from these simulations revealed a y-intercept of 0 in a free diffusing system. However, when diffusion was confined inside closed domains, the y-intercept became positive. They then went one step further and introduced discrete holes in the domains that would allow the diffusing particle to occasionally escape one domain and diffuse into a neighboring domain. The simulation revealed that in this model, as the number of holes in the diffusion barrier increased, the higher the y-intercept value became. This approach has now been used to reveal the existence of multiple modes of diffusion in biological membranes [28,29].

Another variation on the FRAP experiment is fluorescence loss in photobleaching (FLIP). This experiment, like FRAP, relies on irreversible photobleaching, but in FLIP, photobleaching is performed repeatedly on the same cell using the same ROI, and then, fluorescence is measured outside the ROI. This experiment allows for measuring the mobile fraction by observing the amount of fluorescence lost outside the bleaching area since mobile bleached particles will move outside ROI. However, unlike FRAP, FLIP does not reveal diffusion coefficients. 

## 3. Alternative Methods for Measuring Diffusion

### 3.1. Single-Particle Tracking 

FRAP is just one of multiple experimental methods developed to measure diffusion in live cells, each with its own advantages and disadvantages. One such alternative technique is single particle track (SPT). Single-particle tracking was developed in the 1980s by filming gold- or latex-tagged particles using differential interference contrast microscopy [30,31,32]. However, it was not until advancements in fluorescence in the 1990s and early 2000s that this technique flourished [33]. 

SPT uses a high-frame-rate, high-sensitivity camera to follow the diffusion of a single molecule (or a very small number of molecules) over time. Tracing of the particle’s movements over time can then be created from the SPT video, and from that tracing, a variety of parameters can be determined. Today, this imaging is often carried out on a widefield or total internal reflection fluorescence microscope. And recently SPT was performed using photoactivated localization microscopy to achieve subresolution localization of the diffusing particle [34]. 

When performing SPT, proper labeling is a major factor. First, it is necessary to use a tag that will give a high signal-to-noise ratio so that the particle tracking can be accurate. This means that the selected tag must have a high quantum yield, such as a quantum dot. It is also important that labeling is applied so that only a single diffusing particle is attached to the fluorescent label, as to not introduce artifacts into the diffusion data. In addition, to accurately trace a single molecule, the number of fluorescently tagged particles must be kept very low so that separate tracks can be easily distinguished. 

As SPT allows for the direct visualization of the diffusing particle, observations about its diffusion can often be made directly by watching the time-lapse video or tracing the particle’s movements. For example, if the particle is confined within a membrane region or actively transported, these movements can be estimated by simply observing the diffusion track. As every particle is a single data point, SPT allows the identification of subpopulations with distinct diffusion modes. This ability to easily identify subpopulations with unique diffusion characteristics is a great advantage of SPT over ensemble techniques, such as FRAP, where isolating subpopulations is challenging. 

A variety of quantitative measurements can be taken from the SPT tracing, the most common being the mean squared displacement (MSD). MSD is the distance between the starting and stopping location of the particle over a set time frame. Notice that MSD is the direct distance from one point to another, regardless of the path the particle followed to get there. MSD is determined by the rate of diffusion and time by the following equation:

(12) MSD=4Dt

Various diffusion modes can be identified by plotting MSD values at different time intervals against time. If the slope of the line is constant, then the molecule is undergoing free diffusion. However, when the diffusion is confined to distinct domains, and long-range diffusion is constrained but short-range diffusion is not, then MSD vs. time will create a logarithmic growth curve with changes in MSD decreasing as observation times are increased. Finally, if the molecule is actively transported, then the MSD of the line will increase exponentially with time.

### 3.2. Fluorescence Correlation Spectroscopy

Another method for measuring molecular diffusion is fluorescence correlation spectroscopy (FCS). In FCS, a small point of laser light (known as the “waist”) is parked on the plasma membrane. Fluctuations in fluorescence intensity within the waist are then recorded over time. Performing an autocorrelation function on these fluctuations over time can reveal an average retention time that particles spend in the waist, and since the waist size is known, average diffusion rate can be derived. In order for accurate autocorrelation, the fluorescence labeling density must be very low in an FCS experiment to distinguish the comings and goings of single particles from the waist. This places FCS somewhere between a single-particle technique, like SPT, and a true ensemble technique, like FRAP. 

One drawback to FCS is that it has difficulty measuring immobile particles. For example, if a particle remains in the waist for the length of the experiment, it will be discarded as noise in the classic FCS experimental regime. In addition, FCS is traditionally performed with a stationary beam, which can lead to photobleaching of slow-moving or immobile particles. One solution to this problem is to move the beam during data acquisition (a technique known as scanning FCS), which minimizes photobleaching while still allowing for accurate diffusion measurements [35].

However, one great advantage of FCS is that the waist size can be adjusted. In 2005, the labs of Didier Marguet and Pierre-Francois Lenne hypothesized that various modes of diffusion could be identified from FCS data collected over a range of waist sizes since distinct forms of diffusion scale differently with respect to changes in the size of the observation area. By plotting diffusion times from FCS data on the y-axis and the waist area (ω^2^) on the *x*-axis, Lenne et al. showed that they could identify different modes of diffusion on the basis of the y-intercept [36]. For example, molecules that undergo free diffusion or free diffusion around discrete impermeable obstacles would fit a line with a y-intercept of zero. Particles trapped in immobile domains would generate a plot on this graph with a high slope and a negative y-intercept, and particles that dynamically partition into and out of domains or complexes would have a positive y-intercept. Then, by using confocal FCS on a panel of plasma membrane markers, they observed the three modes of diffusion they had predicted [36,37]. Lenne et al. collected their FCS data on a confocal microscope which meant that they could generate waists only above the diffraction limit—approximately 250 nm. But, they predicted that with a small enough waist, the fit line of FCS data for dynamic partitioning or meshwork confined molecules would shift to a y-intercept of zero as the observation area becomes smaller than the partitioning domains or the meshwork [36,37]. 

In stimulation emission depletion microscopy (STED) microscopy, the FCS waist can be reduced theoretically to an infinitesimal spot, although, in reality, STED-FCS is often performed with waists of 20–100 nm. In 2009, Stefan Hell’s group used STED-FCS to measure diffusion in the plasma membrane below the diffraction limit [38]. In this study, when confocal FCS was performed using various waist sizes, the transient time for Atto647N-tagged sphingomyelin did not scale proportionately with waist size and was fit by a line with a positive y-intercept. This positive y-intercept was also present in fitting data collected by STED-FCS with spot sizes above ~80 nm in diameter. However, STED-FCS data on Atto647N-sphingomyelin collected below ~80 nm diameter showed two populations—one with a positive y-intercept (undergoing transient confinement) and one with a y-intercept of zero (undergoing free diffusion). 

Finally, it must be noted that an important variation on FCS, often known as fluorescence cross-correlation spectroscopy (or “FCCS”), has been developed that allows for the simultaneous collection of diffusion information and the detection of molecular complexes [39]. In brief, in FCCS, two classes of particles are labeled with different fluorophores, and both species are monitored simultaneously in the same FCS waist. From the two-color fluorescence intensity data, autocorrelation can be modelled separately on each species to understand each particle’s diffusion. Autocorrelation can also be modelled across the two colors to detect particles diffusing together in a complex [39,40]. 

The ability to perform cross-correlation provides a unique advantage to FCS. Complexes have also been detected by SPT when two labeled species move together as one [41]. For these interactions to be observed, both binding partners must be tagged. This poses a problem because effective SPT requires a very low number of molecules to be labeled at any time. Therefore, the odds of observing these interactions becomes very low, and many of these interactions likely go undetected. Similar attempts have been made to measure co-diffusion with FRAP by simultaneously observing the diffusion of two species labeled with different color fluorophores [42]. However, this technique has not been widely accepted. 

The ability to perform FCS below the resolution limit is a great advantage of FCS and SPT over FRAP, especially given that structures that impact diffusion exist on the order of 100–250 nm, such as clathrin-coated pits, caveolae, and actin corals (which are discussed in detail later). While SPT and FCS can measure diffusion inside and outside these structures separately, FRAP generally reports only an average diffusion across these structures. The ability to measure diffusion below the diffraction limit comes at a steep cost in terms of equipment and training. The conventional SPT and FCS setups require expensive sensors and complex data processing. Additionally, to perform these techniques below the diffraction limit requires an extra equipment investment, which is not feasible for many researchers. Alternatively, the capability to perform FRAP is now standard in most off-the-shelf confocal microscopes and is available to many researchers at major institutions. Furthermore, the *M*_f_ and *D* (derived from *t*_1/2_) are easily calculated and require little mathematical expertise.

## 4. What Diffusion Measurements in Live Cells Have Taught Us about the Plasma Membrane

FRAP is an immensely versatile tool for cell biologists, which has been applied to study a wide variety of biological systems, including the chromatin binding in the nucleus [43], nuclear import/export [44,45,46], intracellular trafficking [47], and turnover of liquid biomolecular condensates [48]. However, its most used application is to study the subresolution structure of the plasma membrane. The diffusion data collected using FRAP, along with SPT and FCS, have been instrumental in advancing our knowledge of the plasma membrane of mammalian cells over the last 50 years. 

### 4.1. The Lipid Raft Hypothesis 

Forming model membranes with a small number of purified lipid species (often phosphatidylcholine, sphingolipid, and cholesterol) in an aqueous buffer can result in a phase separation with a sphingolipid and cholesterol-rich fraction (known as the “Liquid Ordered” or “Lo” domain) and a phospholipid rich fraction (known as the “Liquid Disordered” or “Ld” domain). This observation led to the proposal of a lipid raft model that suggests that sphingolipids and cholesterol self-organize into domains or rafts within the cell membrane [49]. The working model is that these Lo domains are analogous to the rafts that may exist in live cells. 

**Figure 2:**
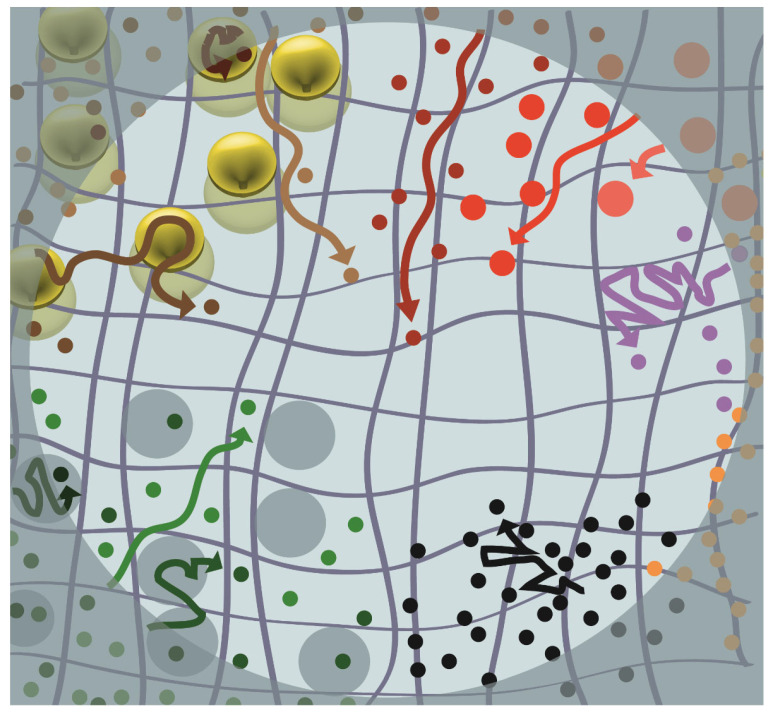
Schematic of some of the models for membrane organization affecting diffusion. Here, diffusion of various molecular species (represented as dots) is conceptualized following a photobleaching event, where the lightly shaded region represents the ROI. The mobility is represented by an arrow, where the arrow length is proportional to the distance traveled over a discrete unit of time. The distance of each arrow from the edge of the ROI to the center is representative of the long-range diffusion that may be observed using FRAP. Among molecules that experience free diffusion (dark red, red, and pink), the Saffman–Delbruck equation states that those molecules with the smallest hydrodynamic radius (dark red) will undergo the fastest diffusion, and those with the largest hydrodynamic radius (pink), the slowest diffusion. Some molecules (purple) are not anchored by actin (dark grey) directly but are transiently confined by cortical actin. Particles anchored to the actin cytoskeleton (orange) experience effectively no diffusion. Proteins (black) can be slowed simply by experiencing a high localized density of other proteins. Molecules that are excluded from rafts (light green) can diffuse around discrete lipid rafts (blue-gray), while diffusion of molecules that are transiently raft-associated (green) or more permanently trapped (dark green) in rafts display slowed diffusion. Likewise, molecules that are trapped (dark brown) or transiently associated (brown) with endocytic pits (yellow) will have reduced long-range diffusion as compared with molecules (light brown) that can freely move around endocytic pits.

As direct visualization of lipid rafts in live cell membranes is rare, rafts are assumed to be subresolution. However, many models have been put forward regarding how rafts may organize the plasma membrane and, in doing so, impact diffusion (Figure 2). One model is that raft components can be trapped in the raft; therefore, they must diffuse as part of the larger raft complex. Alternatively, raft components could become transiently trapped in raft domains before moving on to another raft. Both models suggest that association with delineated rafts will result in slower diffusion than predicted in a homogenous membrane. In one of the most comprehensive studies on this prediction, FRAP was performed on a wide variety of plasma membrane proteins predicted to be raft-associated or not raft-associated [50]. That study revealed that diffusion was more closely aligned with the mode of membrane attachment than a putative assignment to the raft or non-raft membrane domain [50]. This result indicated, at least for these markers, that trapping of proteins in stable rafts is likely not occurring. However, it could not conclusively disprove the existence of rafts, as it is still possible that proteins either move in and out of rafts with ease or that the viscosity of the raft and non-raft factions are too similar to be detected by FRAP. 

Other attempts to detect rafts have relied on perturbations of the membrane that would be predicted to target any raft-associated proteins specifically. For example, the raft hypothesis suggests that removing the cholesterol from the membrane would disperse the rafts. This would allow the raft-associated particles to move freely without a raft, resulting in faster diffusion. The most common method to deplete cholesterol is incubation in methyl-beta-cyclodextrin (MβCD). However, this method has revealed contradictory results; slowing diffusion in some instances and increasing diffusion in others (summarized in [51]). These differences may be due to off-target effects, as alpha-cyclodextran (α-CD), which has a similar structure to MβCD but does not affect cholesterol levels, produced the same effect on membrane diffusion as MβCD when studied side-by-side [52]. An alternative to MβCD is inhibiting cholesterol synthesis by a small molecule. For example, cholesterol reduction using the statin drug compactin increased the diffusion coefficient as measured by FRAP for influenza hemagglutinin (HA) variants that were isolated in the detergent-resistant membrane (putative raft proteins) but did not affect a detergent soluble HA construct [53]. 

One model for lipid rafts proposes that cell membranes have a percolation threshold [54]—a temperature at which the raft goes from being small, interspersed regions to becoming the bulk of the cell membrane, with isolated non-raft domains being the minority of the membrane. In this scenario, it has been proposed that the diffusion coefficient for raft-associated proteins will decrease with decreasing temperature due to increased viscosity but only to a point. Once the temperature is low enough to pass this percolation threshold, the diffusion coefficient of the raft proteins will jump, as they now have the freedom to move not just within the raft but through the cell surface without the confinement of raft boundaries. Data to support this theory comes from FRAP of the putative raft and non-raft markers on the apical membrane of polarized MDCK and Caco-2 cells. At room temperature, the raft markers had fast diffusion and complete recovery after FRAP. In contrast, the putative non-raft markers showed partial recovery and diffusion that was 3-4 times slower than that of the putative raft markers [54].

Interestingly, increasing the temperature to 37 °C resulted in a dramatic increase in mobile fraction for the non-raft markers. From these data, Meder et al. proposed a model where the sphingolipid-rich apical membrane was one large raft with discrete, isolated non-raft domains at room temperature. This restricted the motion of the non-raft marker, resulting in a high immobile fraction. However, increasing the temperature to 37 °C caused the membrane to pass a percolation threshold at which the rafts became isolated domains in a continuous fluid phase. Under these conditions, the non-raft markers could undergo long-range diffusion without being blocked by the raft membrane [54]. However, this dramatic change in diffusion as a function of temperature change has not been reported in non-polarized cells [50,55]. Therefore, the percolation behavior observed by Meder et al. may be unique to the apical membrane containing higher sphingolipid concentrations than non-polarized cells [56]. 

### 4.2. Particle Trapping in Caveolae 

A putative example of a lipid raft regulating cell trafficking is the caveolae, a flask-like plasma membrane invagination identified by the presence of caveolin and cavin proteins [57,58,59,60,61]. These membrane invaginations regulate membrane tension and may facilitate vesicular trafficking [62]. The assembly of these structures is cholesterol and sphingomyelin-dependent [61,63,64], suggesting that they may represent a subclass of lipid raft. Photobleaching of fluorescently tagged caveolin on the cell surface has shown that these microdomains are highly immobile in the membrane plane [65,66]. This immobility is due partly to the caveolin protein crosslinking to actin fibers via interactions with filamin A [65]. In addition, the large size and stability of the caveolin complex could be expected to inhibit lateral movement of the caveolae. The immobility of caveolae suggests that it may trap and immobilize specific membrane components (Figure 2). In our previous study, we tested this theory by FRAP on a panel of membrane markers in caveolin expressing and caveolin knock-out mouse embryonic fibroblasts cells. In that study, we found no difference in the rate of diffusion or mobile faction of the markers tested across the two cell lines among the membrane markers we tested [55]. This result would suggest that caveolae do not trap membrane markers, beyond caveolin and cavin. 

### 4.3. Association with Clathrin-Coated Pits 

Like caveolae, clathrin-coated pits (CCP) docked at the cell surface could potentially influence diffusion (Figure 2). Yoav Henis’s group tested this theory by expressing WT or C543Y mutant influenza hemagglutinin in the monkey fibroblast, CV-1, cell line [67]. WT HA stays at the plasma membrane and is not internalized by clathrin, but the C543Y mutant does internalize through clathrin-mediated endocytosis [68,69]. By performing FRAP on the two markers, they found that WT HA diffuses significantly more quickly than the C543Y variant. The experiment was then repeated under hypotonic conditions (which disassemble CCP at the plasma membrane) and lateral diffusion increased for the C543Y mutant to rates nearly identical to those of the WT protein [67]. This initial study found that only the rate of diffusion, not the mobile fraction, was affected by the CCP association [67]. However, in a follow-up study, HA variants with an approximately 10-fold increase in affinity for CCP were compared to HA-C543Y. Unlike the results in the earlier study, these new variants did show dramatically reduced mobile fractions [70]. Together, these two studies suggest a gradient where non-CCP membrane components have a high rate of diffusion and high mobiles fraction, molecules that are weakly associated with CCP have reduced diffusion coefficients but high mobile fractions, and molecules with a high affinity for CCPs will become trapped in CCPs resulting in reduced diffusion coefficients and low mobile fractions. 

### 4.4. Diffusion Confined by Cortical Actin 

Early FRAP studies suggested that the cytoskeleton near the cell surface may slow diffusion on the plasma membrane [71,72]. These early FRAP results have been advanced by work with single-particle tracking over the last 30 years. Using single-particle tracking, the Kusumi lab observed that the transmembrane domain (TM) of the major histocompatibility complex class II (MHC II) protein (TM-I-E^k^) and a glycophosphoinositol (GPI)-anchored construct of the cell surface domain of MHC II (GPI-I-E^k^) diffuse into a given region for a finite time then quickly move to a new region where it would again become confined for some length of time [73]. Actin disruption with latrunculin A caused the size of the restricted areas to increase, suggesting that cortical actin defines the diffusion barriers observed in the particle tracking [73]. On the basis of this observation, the Kusumi group proposed what is commonly known as the “Picket and Fence” model [74], where some membrane proteins are anchored directly to the cytoskeleton and immobile (i.e., “pickets”). These actin-immobilized pickets may account for up to 30% of all transmembrane proteins [74]. Cortical actin anchors these pickets, which become a diffusion barrier (i.e., “fences”) to other membrane particles. Single-particle tracking suggests that actin-delineated regions are typically ~230 nm across [74]. Inside these domains proteins display Brownian diffusion, but occasionally a trapped molecule will escape one corral and become confined in a new corral (Figure 2). This hopping between corrals will present itself as sub-diffusion over larger spatial distances. 

### 4.5. Effects of Membrane Line Tension 

In addition to molecular diffusion, cortical actin regulates lateral membrane tension displacement. Localized changes in membrane tension have been implicated in many cellular processes, including exocytosis [75], and endocytosis [76,77], migration [75,78], mitosis [79], and signaling [78,80]. However, it is unclear whether localized tension changes extend to the cell membrane’s distal regions. Shi et al. tested the hypothesis that localized changes in membrane tension would propagate to the entire plasma membrane in a set of elegant experiments in *Cell* [81]. Using micropipettes, Shi et al. pulled two membrane tethers on opposing sides of the cell. By further pulling or relaxing the tethers, they were able to measure how changes in pressure in one tether altered pressure in the other tether. From this, they could determine the degree and rate of long-range displacement of changes in membrane tension. They then used FRAP to determine the fraction of immobile membrane proteins and membrane viscosity. Combining the FRAP data and the results of the tethering experiment, a long-range tension diffusion coefficient of 0.0024 μm^2^/s was derived [81]. Interestingly, this tension diffusion coefficient was 100–1000-fold slower than typical molecular diffusion. When tension measurements were taken in giant uni-lamellar vesicles or cytoskeleton-free giant membrane vesicles, quick long-range displacement of tension was observed [81,82], suggesting that the cytoskeleton plays a primary role in restricting tension displacement. 

### 4.6. Effects of Protein Density on Diffusion 

Lastly, protein density may impact diffusion as having a high number of large membrane protein in a small space may lead to crowding (Figure 2). Frick et al. observed this effect utilizing brefeldin A to block the transport of newly synthesized proteins to the plasma membrane. As expected, this treatment reduced membrane protein levels significantly, as quantified by staining with an anime-reactive dye. Using FRAP, they showed that reducing plasma membrane protein levels leads to a significant increase in the diffusion rate for GFP-GT46, an artificial transmembrane protein. Therefore, it was concluded that the crowding of proteins caused by a high protein density in the plasma membrane could slow diffusion [83]. However, brefeldin A inhibits vesicular trafficking from the Golgi and, in doing so, affects both the plasma membrane’s protein and lipid composition [84,85,86,87]. The observed increase in diffusion following brefeldin A treatment, therefore, may not solely be the result of altered membrane protein levels, although a reduction in protein density does appear to be, at least in part, involved in regulating diffusion. 

## 5. Conclusions 

As the field of membrane biology has progressed it has uncovered ever more complexity in the structure and organization of the cell membrane. Many factors that organize the cell membrane have been identified. But the work is far from done. More structural features of the plasma membrane are likely to be discovered. And with all that we have learned about the plasma membrane since Frye and Edidin [1] first demonstrated the fluidity of the plasma membrane over 50 years ago, it is still not fully understood how the various factors that regulate membrane microstructure (such as rafts, actin domains, protein density, and endocytic pits) work together to support all of the functions of the cell membrane. To date, diffusion measurements have been invaluable in elucidating the organization of the cell membrane. And, FRAP has proven to be an important method to collect diffusion data from live cells. Given its versatility and accessibility to the research community it should remain an important technique in future membrane biology research.
